# Noncontact optical 3D strain measurements in cervical soft tissues biomechanics by digital image correlation under tensile test: an experimental approach

**DOI:** 10.3389/fbioe.2025.1493476

**Published:** 2025-03-11

**Authors:** Fangzheng Lin, Yaoqian Cai, Jing Li, Jiheng Zhan, Zibo Gao, Xiaolong Zeng, Minshan Feng, Yongjin Li, Dingkun Lin, Ji Qi

**Affiliations:** ^1^ Wangjing Hospital of China Academy of Chinese Medical Sciences, Beijing, China; ^2^ Key Laboratory of Beijing of TCM Bone Setting, Beijing, China; ^3^ The Second Clinical Medical College, Guangzhou University of Traditional Chinese Medicine, Guangzhou, China; ^4^ The Second Affiliated Hospital, Guangzhou University of Traditional Chinese Medicine, Guangzhou, China; ^5^ Department of Orthopedics, Guangdong Provincial Hospital of Traditional Chinese Medicine, Guangzhou, China; ^6^ Guangzhou Key Laboratory of Cervical Mechanobiology, Guangzhou, China; ^7^ Chinese Medicine Guangdong Laboratory, Zhuhai, China

**Keywords:** digital correlation imaging, cervical spine, *in vitro*, tensile test, biomechanics

## Abstract

**Background:**

Digital image correlation (DIC) is widely used to measure surface strain in loaded objects. When studying the deformation of the cervical spine, the complexity and non-planarity of the structure complicate the speckle pattern required for applying DIC. While this non-invasive technique has shown promise in biomechanical testing, its application to cervical spine analysis presents unique challenges, particularly in achieving dynamic full-scale multi-aspect strain measurements. The aim of this paper is to introduce a method for exploring the stress-strain relationship on cervical cadaveric specimen by optical non-contact measurement system.

**Method:**

Cervical cadaveric specimens were selected as subjects. Before testing, anatomical exposure, embedding, and spraying were performed sequentially. Specimen preparation was optimized through transverse process removal to enhance visualization of key anatomical structures. The surface strain under tensile testing was analyzed by the Aramis non-contact measurement system.

**Result:**

High-quality three-dimensional strain images were obtained with improved inspection points across all aspects, particularly in the lateral aspect (5397.25 ± 723.76 vs. 3268.25 ± 573.17, *P* < 0.05). Under 60N tensile loading, strain distribution revealed concentration in soft tissue regions while preserving clear visualization of vertebral bodies, intervertebral discs, and foramina. Quantitative analysis shown consistent deformation patterns across cervical segments (C4-C7), with no significant differences in segmental parameters (*P* > 0.05).

**Conclusion:**

The application of an optical non-contact measurement system in this study of cervical spine biomechanics has been proven effective. This method potentially mitigates some of the limitations associated with previous DIC techniques when applied to cervical cadaveric specimens. As a result, it enables more available measurements of multidimensional strain, which may enhance our understanding of the mechanics of the cervical spine.

## 1 Introduction

In recent decades, collaboration between orthopedics and biomechanics has grown significantly. Within this progress, *in vitro* mechanical testing on cadaveric specimens has become a critical component of orthopedic research. It could provide helpful experimental evidence for clinical practice, such as the way of internal fixation, the effects of mechanical environment on bone healing, the safety of non-surgical treatment, and so on (Le et al., 2015; Ettinger et al., 2018). Regardless of the type of test—compression, torsion, traction, or specific load—each requires the materials to undergo observed strain and deformation. Generally, strain is used to estimate by the extent of deformation in materials. Conventional approaches include strain gauge, linear variable differential transformers, fiber bragg grating (Grassi and Isaksson, 2015). However, these measurement methods present several disadvantages. First, they are costly and cumbersome to operate, with their accuracy significantly influenced by external environmental conditions such as temperature and moisture. Second, methods like linear variable differential transformers, strain gauges involve manual sensor attachment and or only provide localized response values, which hinders the evaluation of global mechanical behaviors. While finite element analysis can provide overall strain distribution predictions, it relies on idealized models and assumptions that may not fully capture the complex behaviors of actual biological tissues, thus cannot serve as a complete substitute for *in vitro* biomechanical testing. Third, the invasive nature of inserting sensors to measure internal strain can alter mechanical behaviors (Brandolini et al., 2014; Danesi et al., 2016). Consequently, there is a demand for non-contact strain measurement techniques in biomechanical applications.

Digital image correlation (DIC) is an optical method that utilizes one or multiple cameras to quantify surface strains on an object subjected to loading. In the early 1990s, the integration of DIC with stereovision led to the development of three-dimensional (3D)-DIC, a non-contact strain measurement technique using multiple cameras to capture the 3D positions on objects. The basic principle of the method is to find the image correlation between a reference image and a deformation image under different load levels (Lucas, 1981). By storing digital images, it is possible to measure the displacement and strain fields in the region of interest based on greyscale similarity (Schreier et al., 2009). This advanced technique has been applied in fields ranging from aerospace (Sutton and Michael, 2003), micro-scale measurements (Schreier et al., 2004; Sutton et al., 2008) to biomechanics of spine and joints (Dickinson et al., 2011; Cano-Luis et al., 2012; Holsgrove et al., 2015). However, its application in cervical spine biomechanics has revealed several challenges.

These challenges can be categorized into two main aspects: technical limitations and specimen-related factors. While technical limitations such as camera resolution, shooting speed, noise, calibration errors, and software algorithms remain largely fixed constraints (Pan, 2018), specimen-related factors present variable challenges that warrant particular attention. The cervical spine’s complex anatomical structure introduces unique difficulties in DIC application. The primary challenge lies in the cervical spine’s complex and non-planar nature. Its multiple overlapping structures, including transverse processes, vertebrae, and ligaments, create optical path obstructions that affect measurement quality.

Previous studies have attempted to address these challenges with varying approaches. Liu (Liu et al., 2014) demonstrated the effectiveness of DIC in measuring strain distribution in porcine IVD under unconfined compression and tension loads. Yang (Yang et al., 2022) found that the 3D-DIC system can accurately assess the radial bulging and axial strains of porcine IVD during creep. However, most studies have predominantly focused on animal specimens or short-segment human specimens (Causa et al., 2002; Spera et al., 2011; Holsgrove et al., 2015), with observations primarily confined to single-segment analysis and single-aspect imaging, limiting our understanding of the cervical spine’s integrated biomechanical behavior. And so on, the complex structures complicate the application of speckle patterns necessary for DIC and impacts image quality, which can lead to inaccuracies in strain measurements, and it is a critical concern in biomechanical studies (Cristofolini, 2017). Therein, cervical soft tissues such as intervertebral disc (IVD) and ligamentum flavum (LF) are affected by these structural complexities, which also extend to other crucial structures like the intervertebral foramen (IVF). These structures are essential for understanding the mechanical mechanisms underlying various spinal diseases and corresponding interventions (Anderst et al., 2016; Wong et al., 2022). Furthermore, intricacy affects the application of DIC in the dynamic loading of cervical cadaveric specimens (such as flexion, extension, torsion, and traction), potentially limiting its utility in certain *in vitro* biomechanical experiments.

These technical challenges in measuring soft tissue response become particularly relevant in the context of cervical traction therapy, a well-established conservative treatment for chronic cervical disorders (Blanpied et al., 2017; Graham et al., 2008). While this non-invasive method has demonstrated significant clinical efficacy in symptom management through controlled tensile forces that increase intervertebral space and neural foraminal area (Côté et al., 2016; Zhu et al., 2016), the underlying biomechanical mechanisms, particularly regarding soft tissue response during traction, remain inadequately understood.

Therefore, this paper aims to address these challenges by presenting an experimental approach for preparing cervical cadaveric specimens and applying DIC to measure strain and deformation during *in vitro* tensile testing.

## 2 Materials and methods

### 2.1 Ethics

All procedures performed in this study involving human participants followed the Declaration of Helsinki (as revised in 2013). The procedures were approved by the Ethics Committee of Guangdong Provincial Hospital of Chinese Medicine. The donors have dedicated their bodies for educational and research purposes to the local Institute of Anatomy prior to death, in compliance with local institutional and legislative requirements.

### 2.2 Materials

A total of four resh frozen cervical cadaveric specimens (C0-T1) were visually selected and examined by X-ray to rule out bone abnormalities such as tumor, fracture, dislocation, deformity, and severe osteoporosis. These specimens, ranging in age from 38 to 52 years, were preserved from the occipital bone to the T1 vertebra. All specimens were donated voluntarily, and approved and kept by the Department of Anatomy, Southern Medical University.

### 2.3 Specimens preparation

The cervical cadaveric specimens were carefully dissected, removing all soft tissues (skin, muscles, nerves, and blood vessels) while preserving the bony structures, ligaments, and joint capsules. The transverse processes were removed to clearly expose the intervertebral foramen (IVF), facilitating anatomical visualization and experimental measurements. After the dissection, specimens were wrapped in paper towel, sprayed with 0.9% saline solution, triple sealed in plastic bags, and then stored at −24°C.

On the morning of testing, each specimen was left to thaw for 6 h at room temperature (20°C ± 2°C). Four screws (2 mm diameter) were inserted into specific locations: the cerebellar fossae of the occipital bone, T1 vertebra, and T1 spinous process. Additional screws were added when necessary to enhance the specimen-potting block interface. To stabilize the highly mobile atlantoaxial joint, 1.2 mm Kirschner wires were bilaterally inserted from C1 to C2 transverse processes. Then, the specimens were potted in an embedding mold using a denture base resin mixture (powder: solvent = 1:1 ratio, total 200 mL), solidified naturally to hold the specimen securely during the process. Special attention was given to keep the cranial and caudal ends aligned, ensuring accurate mechanical testing.

### 2.4 Application of speckle pattern

**FIGURE 1:**
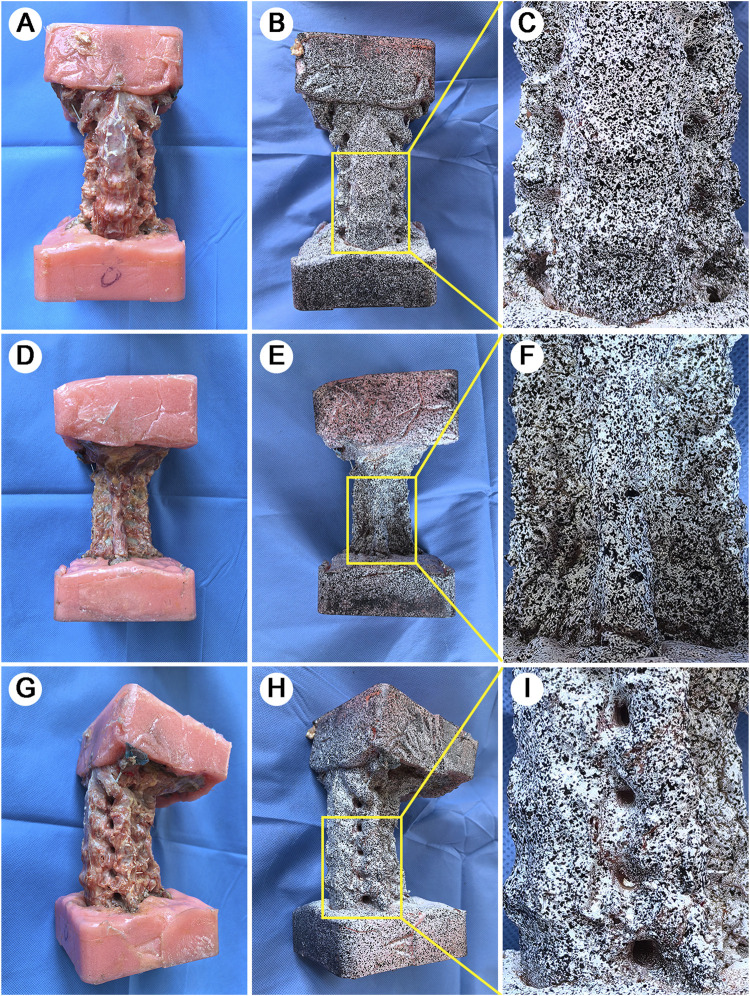
Specimen preparation and speckle pattern application. A full-scale view of specimen preparation across multiple anatomical aspects. **(A)**: Anterior view of the potted specimen showing standardized embedding technique. **(B)**: Anterior aspect after white base coat and black speckle pattern application. **(C)**: Magnified view of vertebral-disc interface highlighting pattern detail and anatomical definition. **(D)**: Posterior view after soft tissue removal showing vertebral plate. **(E)**: Posterior speckle pattern application. **(F)**: Magnified view of spinous process and lamina complex with applied pattern. **(G)**: Lateral view following TP removal for enhanced DIC 3D visualization. **(H)**: Lateral speckle pattern showing clear delineation of IVF. **(I)**: Magnified view of vertebra-disc-articular process complex demonstrating pattern integrity across different tissue types.

For Digital Image Correlation (DIC) measurements, specimens were first dried and then painted with a white base coat. The black speckle pattern was then applied separately to each surface (anterior, left lateral, right lateral, left posterior arch, and right posterior arch) using an airbrush technique. During the application process, adjacent surfaces were masked to ensure the randomness of the speckle pattern and maintain high black-white contrast on each surface (Figure 1). This systematic method to speckle pattern application ensures optimal conditions for DIC tracking of surface displacements and strains.

### 2.5 DIC system setup and tensile test

**FIGURE 2:**
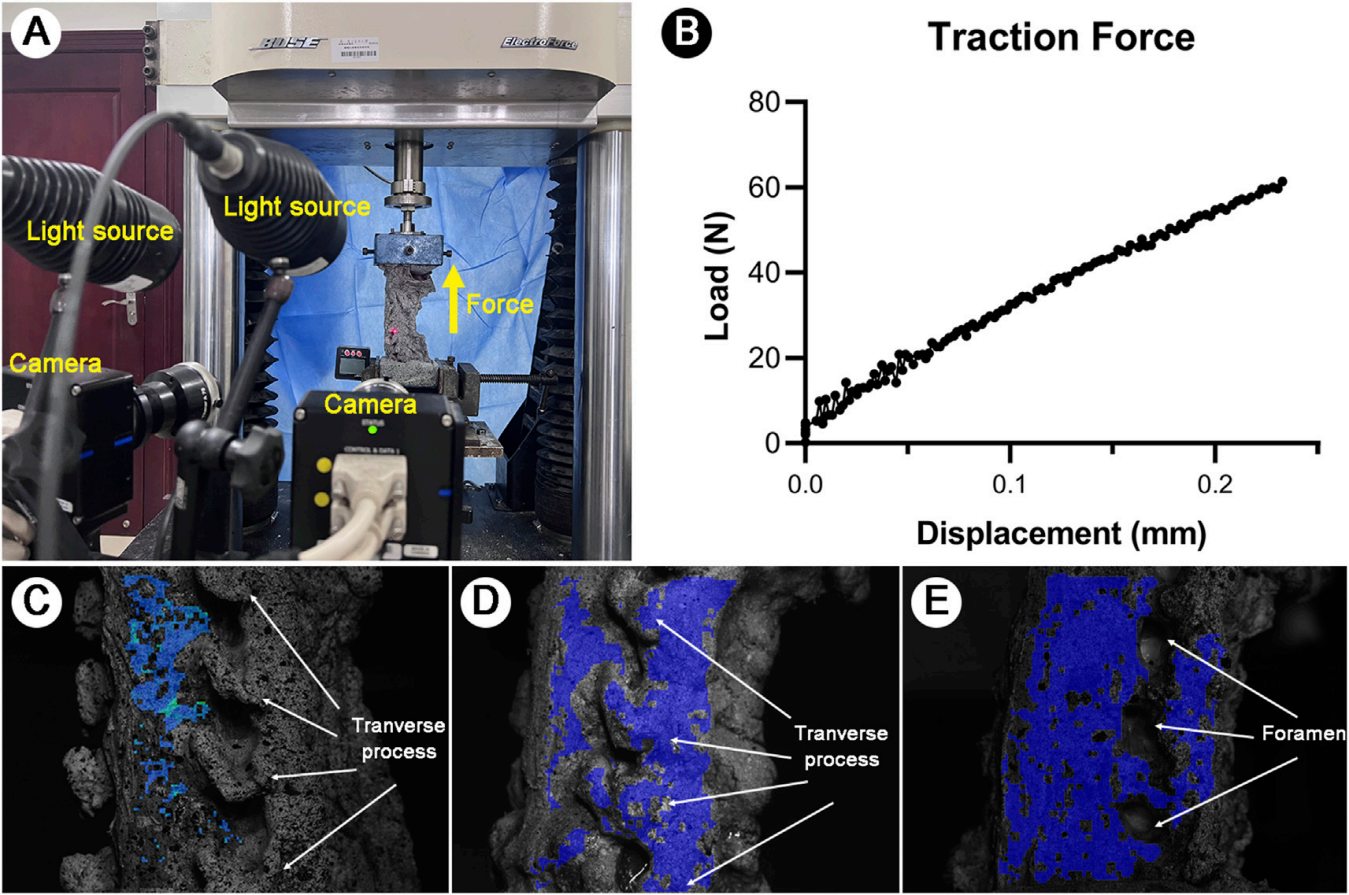
Experimental setup and methodological optimization. **(A)**: Integrated testing configuration showing servo-hydraulic system and dual-camera DIC setup. **(B)**: Loading protocol diagram illustrating the 60N progressive axial tensile force application. **(C)**: Focus interference caused by the overlapped TP and inadequate 3D inspection points. **(D)**: The overlapping of the TP caused 3D inspection points were identified only on the TP and articular facets, but it was failure to measure the IVF. **(E)**: Optimized lateral view following transverse process removal. Clear visualization of vertebral bodies, IVF and sufficient 3D inspection points for 3D SS modeling during tensile testing.

The cervical specimens were mounted on the servo-hydraulic material testing system (Bose Electro Force 3520-AT; Bose, MN, United States). A non-contact optical 3D strain measuring system (Aramis 3D camera 6M, GOM, Braunschweig, Germany) was used to record specimen deformation and strain during testing (Figure 2A).

The dual-camera system was calibrated by adjusting camera heights and distances until optimal image clarity was achieved. For anterior view measurements of C4-C7, cameras were centered on the C5 vertebral body, ensuring coverage from C4 lower endplate to C7 upper endplate. Similar setups were applied for lateral and posterior views. This configuration enabled full-scale imaging of the IVD, IVF, and lamina range C4-C7. Images were captured at 4 frames/second with a resolution of 2,448 × 2,050 pixels and a displacement precision of 0.001 mm.

Biomechanical testing was conducted using Win Test Digital software. Laboratory temperature was maintained at (20°C ± 2°C) throughout the testing process. Prior to the tensile test, 4-5 static images were captured by the Aramis system to verify focus quality and speckle pattern integrity. The measurement module was opened to select 3-5 initial inspection points on each target area such as C4/5 IVD, C5/6 IVD, C6/7 IVD. A minimum of 4,000 3D inspection points were required across anterior, lateral, and posterior aspects of C4-C7. Remarkably, the removal of the TP eliminated previous challenges related to focus interference and overlapping of the IVF, which allowed for clear contour imaging and precise measurement of the IVF structure (Figures 2C, D, E).

Between each test, specimens remained mounted but completely unloaded, with 2-min intervals between tests. Specimens were kept moistened throughout testing. If surface fluid seepage or paint deterioration was observed, the affected areas were promptly dried and repainted. Testing commenced with the activation of the Aramis strain measurement system. Each specimen was subjected to a 60N axial tensile force (Jellad et al., 2009) followed by immediate release (Figure 2B).

### 2.6 Measurements

#### 2.6.1 3D inspection points

The number of 3D inspection points was calculated by the measurement module of Aramis system.

#### 2.6.2 3D Surface strain

3D Surface Strain (SS) characterizes the mechanical response of specimens under tensile testing by measuring the relative displacement between tracked points on the specimen surface. In this study, major strain was defined as longitudinal strain. All 3D SS values represent maximum measurements obtained at a 60N tensile load, comprising both global SS and local SS.

**FIGURE 3:**
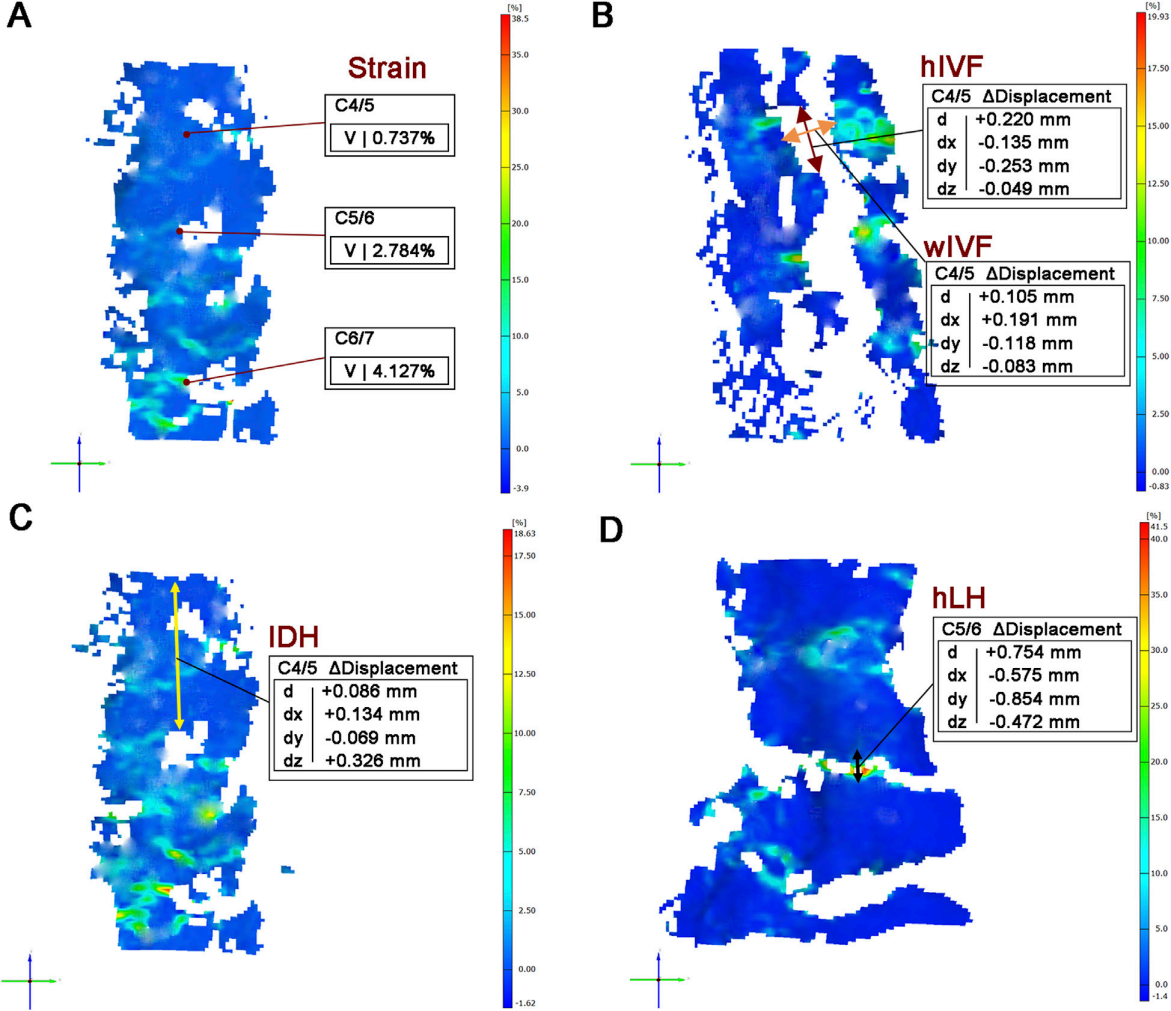
Quantitative measurement parameters for biomechanical analysis. **(A)**: The local SS values of IVD. **(B)**: Measurement of hIVF and wIVF at C4/5 level. **(C)**: Measurement of IDH at C4/5 level. **(D)** Measurement of LH at C5/6 level. Note: V: values; d: displacement XYZ; dx: displacement X; dy: displacement Y; dz: displacement Z.

Global SS values were calculated by the Aramis system and presented in 3D visualization. Local SS values were specifically focused on the central region of the anterior IVD surface for analysis (Figure 3A).

#### 2.6.3 Intervertebral disc height

IDH is measured as the distance between the midpoint of the upper endplate of the target segment and the midpoint of the lower endplate of the adjacent segment, using a lateral cervical radiograph (Jin et al., 2021). The change in IDH (ΔIDH) is then calculated to quantify the disc height alteration before and after axial tensile test (Figure 3C).

#### 2.6.4 Height of intervertebral foramen and width of intervertebral foramen

The hIVF is determined by the distance between the superior medial point and the inferior medial point of the foramen. The wIVF was measured by calculating the distance from the anterior medial point to the posterior medial point of the foramen (Sun et al., 2021). The change in height (ΔhIVF) and width (ΔwIVF) captures alterations pre- and post-axial traction (Figure 3B).

#### 2.6.5 Height of and lamina height

Measurement of LH at the posterior side of cervical cadaveric specimen. The hLH was defined by selecting the lower edge of the upper lamina and the upper edge of the lower lamina, measured at the midpoint of each segment’s lamina (Park et al., 2022). △hLH determined the change in pre- and post-test in axial (Figure 3D).

### 2.7 Statistical analysis

The sample size was calculated using the number of 3D inspection point at lateral side as the primary efficacy parameter. Based on the result of preliminary experiment and related literature, a sample size of 3 specimens per group was estimated to provide 90% power to detect a paired between-group difference of 2000, assuming a standard deviation of 5000, and two-sided significance level of 5%. To compensate for a 25% failure to biomechanical tests, the sample size was increased to 4 specimens in each group. Variables such as the number of 3D inspection point, maximum SS (%), SS of the IVD (%), △IDH (mm), △hIVF (mm), and △hLH (mm) were expressed as mean ± standard deviation. To compare the differences in inspection point between approaches, paired t-test was analyzed. Difference in SS under tensile test among the anterior, lateral and posterior sides, and differences in SS of the IVD (%), △IDH (mm), △hIVF (mm), and △hLH (mm) between segments, were performed by analysis of variance of randomized complete block design. All statistical analysis was conducted using SPSS software (version 20, IBM Corp). All statistical tests were two-sided, and the threshold for statistical significance was set at *P* < 0.05.

## 3 Results

### 3.1 3D inspection points distribution

**FIGURE 4:**
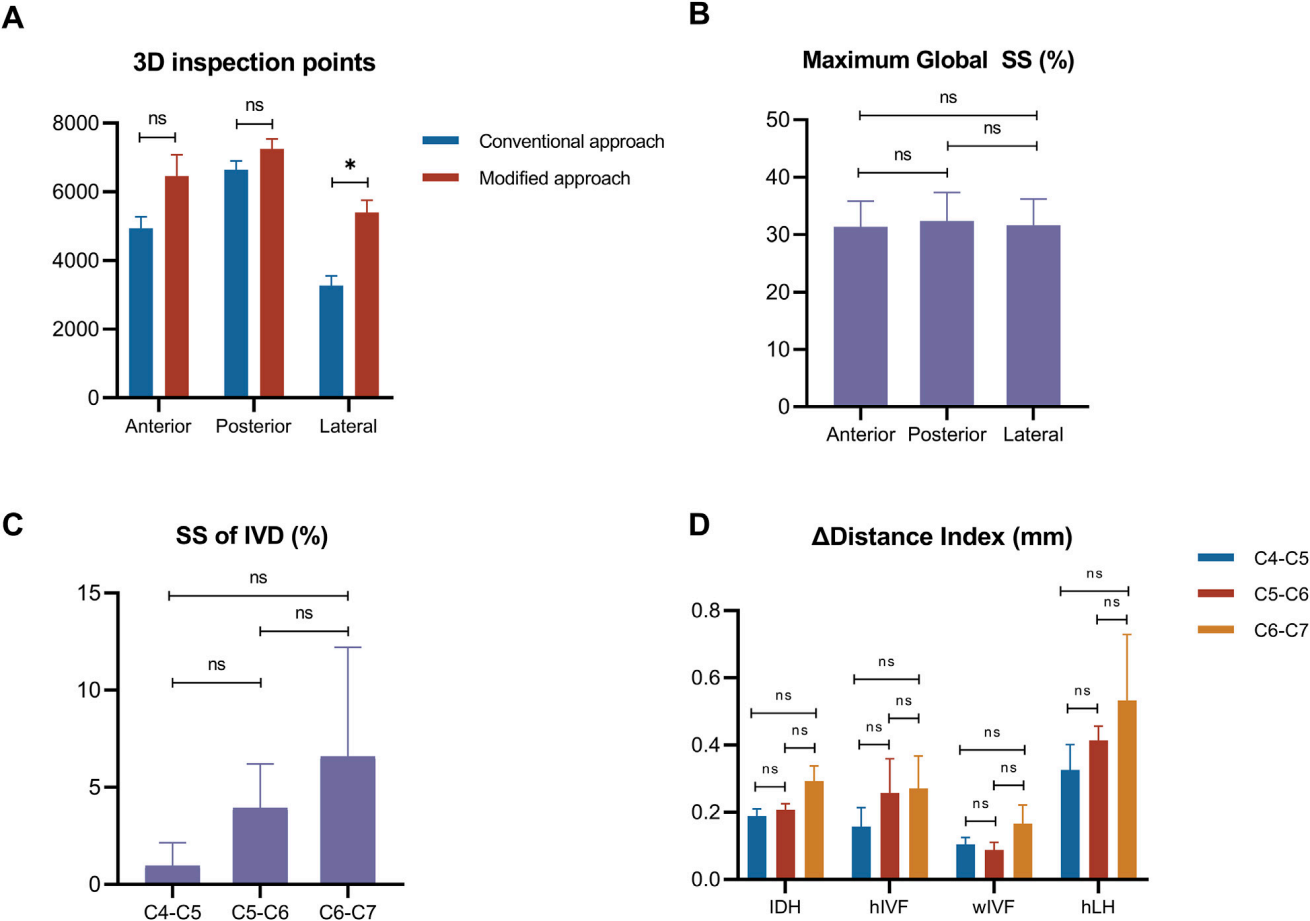
Distribution of 3D inspection points and parameter changes across segments. **(A)**: Comparison of 3D inspection point numbers between conventional and modified methods across different aspects. **(B)**: Maximum global SS comparison between aspects. **(C)**: Segmental analysis of local SS of IVD. **(D)**: Combined analysis of dimensional changes. Notes: *P < 0.05; ns = none significant difference.

In the lateral side, the number of 3D inspection points under the modified method, increased significantly, compared to the origin method (*P* < 0.05) (Table 1). Meanwhile, no significant difference was found in the anterior and posterior side (*P* > 0.05) (Figure 4A).

**TABLE 1:** The number of 3D inspection points by different approaches.

Aspect	Conventional approach	Modified approach	*t*	*P*
Anterior	4,936.25 ± 666.77	6,460.00 ± 1241.16	2.947	0.060
Posterior	6,647.00 ± 502.16	7,247.00 ± 597.73	2.211	0.114
Lateral	3,268.25 ± 573.17	5,397.25 ± 723.76	5.709	0.011*

*P < 0.05.

### 3.2 3D surface strain

**FIGURE 5:**
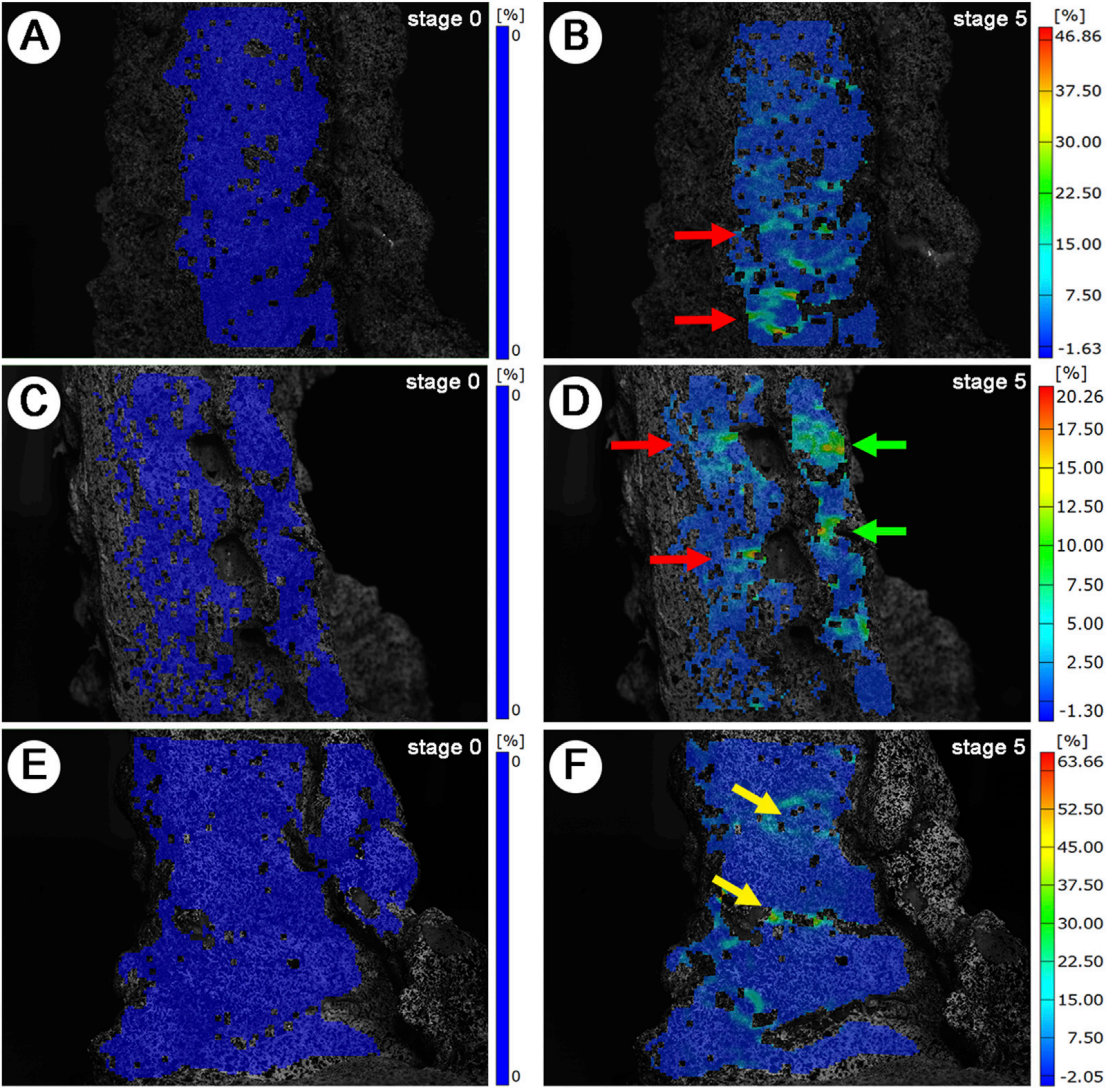
3D Strain Distribution Under Tensile Loading. **(A, C, E)**: Baseline (0N) strain distribution. **(A)**: Anterior view. **(C)**: Lateral view. **(E)**: Posterior view. **(B, D, F)**: Maximum load (60N) strain distribution. Red arrows: IVD SS concentrations. Green arrows: Lateral IVD SS and articular process SS concentrations. Yellow arrows: Laminar space SS concentrations.

Following the described preparation method for cervical cadaveric specimen, successful generation of 3D SS models of the anterior, lateral, and posterior aspects was achieved. The inspection points are densely distributed and uniform, clearly delineating the 3D contours of the cervical cadaveric specimens. The imaging distinctly displays the structural hierarchy of the vertebral bodies, IVD, IVF, articular processes, and lamina (Figures 5B, D, F) under maximum loading (60N), in comparison with the baseline (0N) (Figures 5A, C, E).

During the tensile test, the maximum global SS values were around 30%, without significant difference found among the anteror, posterior and lateral side (*P* > 0.05) (Figure 4B). The SS of IVD (%) showed an increasing trend from C4-C5, C5-C6 to C6-C7, indicating higher SS of IVD in lower segments, but there were no significant differences in the SS of IVD (*P* > 0.05) (Figure 4C).

### 3.3 Deformation of IDH, IVF and LH

The results of deformation of △IDH, △IVF and △LH showed increasing trends from C4-C5, C5-C6 to C6-C7, suggesting greater deformation in lower cervical segments under tensile load, but there were no significant differences in the increase of deformation (*P* > 0.05) (Figure 4D).

## 4 Discussion

The present study investigated the application of DIC technology for examining cervical soft tissue biomechanics through non-contact optical strain measurements under tensile loading conditions. This investigation focused on developing and validating an experimental approach for cervical cadaveric specimen preparation and testing methodology that would enable reliable 3D SS analysis across multiple anatomical aspects of the cervical spine. The experimental method integrated traditional biomechanical testing methods with optical measurement techniques, allowing for simultaneous assessment of various structural parameters during tensile loading. This technical framework may contribute to our understanding of cervical spine biomechanics through non-contact measurement methods.

### 4.1 DIC technology

DIC is a non-contact experimental method for storing images of material surfaces in digital form (Yoon et al., 2021). The system captures images at predetermined intervals, which are analyzed to determine displacement and strain distribution across the disc surface. This capability is particularly valuable in the context of cervical spine biomechanics, where understanding the strain distribution during dynamic loading conditions can inform surgical interventions and rehabilitation protocols.

Initially, the technique was only capable of measuring a single plane. With the development of imaging technology, the application of 2 or more high-speed camera imaging enables the detection of 3D disembodied space (Grassi and Isaksson, 2015; Yoon et al., 2021). DIC technology was first applied to the testing of engineering materials and is now used more in biomechanical testing in dentistry and orthopedics (Bay, 1995; Lu and Cary, 2000; Poissant and Barthelat, 2010).

The technology has the following advantages (Grassi and Isaksson, 2015): (1) Sensor technology alters the displacement or deformation information of an object, DIC is a non-contact method and provides a richer set of discrete data points compared to sensing technology; (2) DIC technology is faster to analyze, cheaper and easy to integrate and consolidate with a few other technologies. However, the accuracy of DIC technology is based on high-quality imaging, which is affected by hardware, software, and the material itself.

### 4.2 Specimen preparation

Given these technical characteristics of DIC, careful attention must be paid to specimen preparation to ensure measurement accuracy, particularly when applying this technology to complex anatomical structures like the cervical spine. On irregular anatomical structures such as spine and joints, which do not have a flat surface like materials such as teeth and endothelial plants, preparation of specimen and speckles spraying becomes more difficult and inefficient. Xu (Xu et al., 2018)successfully detected the strain field of the inferior tibiofibular coalition ligament using the DIC technique, which was also used in the author’s team’s previous study to achieve imaging and detection of the sacroiliac joint (Li et al., 2022).

The complexity of cervical spine presented significant challenges for DIC visualization. It was imperative to ensure that specific areas like IVD, IVF and LF were sufficiently flat to maximize 3D inspection points. A particular challenge emerged in the lateral aspect observation, where protruding TP affected speckle pattern application and obstructed camera focus, hindering strain detection of the IVD and IVF. Our solution involved careful excision of the cervical TP, which preserved bony structure stability while significantly improving visualization of key anatomical structures. This modification enabled precise measurements of IVF and enhanced strain detection across IVD, LF, and articular processes.

Tissue hydration management emerged as another critical consideration due to the high-water content of cervical soft tissues, particularly the IVD. To maintain normal physiological properties and mechanical performance (Causa et al., 2002), specimens were wrapped in wet towels and sealed in plastic bags before speckle pattern application. During testing, when surface fluid seepage occurred, affected areas were carefully dried and speckle patterns reapplied to ensure measurement accuracy. Through this optimized preparation protocol, we successfully monitored strains and deformations across multiple anatomical planes of C4-C7 during dynamic tensile testing. The instantaneous loading protocol (0–60N) employed in this study significantly minimized the risk of tissue dehydration during testing. Furthermore, while tissue hydration is crucial for maintaining physiological properties, it is noteworthy that previous biomechanical studies have rarely provided detailed documentation of environmental conditions and specimen humidity control (as shown in Table 2).

**TABLE 2:** Comparison of DIC applications in spinal biomechanical studies.

Specimen	Number of specimens	Type of mechanical test	Aspect of observation	Type of speckle pattern	Strain ponits	DIC system	Reported quantities	References
Human lumbar (L3/4)	14	3 directions of motion	Lateral of IVD	N.A	N.A	N.A	Strain	Wangsawatwong et al. (2023)
Porcine cervical (C2-C7)	10	Compression	Anterior of IVD	N.A	N.A	VIC-3D	Deformation and strain	Yang et al. (2022)
Human pelvic	6	compression, tensile, rotation	Lateral	Airbrush	N.A	Aramis 6M	N.A	Li et al. (2022)
Porcine cervical (C2-C6)	8	Compression	Anterior of cervical (C2-C6)	N.A	Each specimen equating to approximately 3,000 inspection points in the entire anterior aspect	Vic-3D	Deformation and strain	Holsgrove et al. (2015)
Human lumbar (L5)	1	N.A	Anterior of vertebra	Airbrush	N.A	Dantec Dynamics	Strain	Palanca et al. (2015)
Porcine lumbar (Single segment)	1	Compression	Anterior, posterior, lateral of IVD	Powder	N.A	Dalsa falcon	Deformation and strain	Spera et al. (2011)
Human cervical	4	Tensile	Anterior, posterior, lateral of cervical (C4-C7)	Air brush	Each specimen had an average of 6460, 5397, and 7247 inspection points on the anterior, posterior and lateral, aspects	Aramis 6M	Deformation and strain	Our study

Notes: References are listed in chronological order within each subsection.

N.A = information not available.

### 4.3 Application of speckle pattern

The effectiveness of DIC technology heavily relies on the uniform application and quality of speckle patterns across the entire area of interest. While existing literature provides general guidance on speckle pattern optimization, there remains no standardized protocol for speckle application on cervical cadaveric specimens. According to Quino’s study (Quino et al., 2021), the optimal speckle size is typically determined based on the resolution of the imaging system and the expected deformation of the material being tested. Larger speckles may be appropriate for materials undergoing significant deformation, while smaller speckles are more suitable for materials with minimal movement (Cariero et al., 2014).

In our study, we first validated the collection of sufficient 3D inspection points through static image capture before proceeding with tensile testing. The results demonstrated remarkable improvements in data collection density, with the anterior aspect detecting (6460.00 ± 1241.16) inspection points, the posterior aspect (7247.00 ± 597.73) points, and the lateral aspect (5397.25 ± 723.76) points. These results significantly exceed and more comprehensive than the previously reported threshold of approximately 3,000 points from the anterior aspect by Holsgrove (Holsgrove et al., 2015), indicating a substantial enhancement in measurement resolution. This higher density of inspection points carries several critical implications for biomechanical analysis. First, it enables the construction of multi-aspect and accurate 3D SS model. Second, it allows for precise measurement of strain values at each inspection point, particularly crucial for analyzing complex anatomical interfaces such as IVD and IVF. Furthermore, this enhanced resolution facilitates more accurate measurements of 3D vertebral angles and strain directions, providing a more complete understanding of cervical spine biomechanics.

Our review of existing literature reveals that DIC applications in spinal biomechanics have been relatively limited, particularly regarding multi-aspect and multi-segment strain analysis (Table 2). Most previous studies focused on single aspects (anterior or lateral) or individual segments of the spine, primarily using porcine models. Our study extends this scope by simultaneously analyzing anterior, posterior, and lateral aspects across multiple cervical segments (C4-C7) in human specimens, potentially providing a more comprehensive understanding of cervical spine biomechanics under tensile loading conditions.

Additionally, regarding speckle application methods, several techniques such as airbrushing, spin coating and powder, have been developed for DIC analysis, as documented by Dong (Dong and Pan, 2017). Maintaining consistent gray intensity distribution before and after deformation is crucial for accurate image correlation (Yoon et al., 2021). While powder-based methods offer precise control over speckle dimensions, they often struggle with pattern adhesion during mechanical testing, particularly problematic for hydrated tissues (Dong et al., 2015; Jonnalagadda et al., 2010). The airbrush method, in contrast, demonstrates superior control over pattern characteristics and has been extensively validated in biomechanical studies (Ottenio et al., 2015; Palanca et al., 2015; Zhang and Arola, 2004). This technique allows for better management of pattern density and distribution, providing more uniform coverage and enhanced pattern stability during deformation (Lionello and Cristofolini, 2014). Its versatility in accommodating different specimen sizes has made it a preferred choice in numerous soft tissue studies (Lauret et al., 2009; Yamaguchi et al., 2011; Zhang and Arola, 2004).

### 4.4 Dynamic test

Few studies have mentioned DIC technology may not be suitable for dynamic testing conditions where rapid loading or unloading occurs (Lisický et al., 2022). It is because the DIC technology can be limited by the need for high-resolution imaging and sophisticated software for data analysis. However, Timothy (Holsgrove et al., 2015) has demonstrated the viability of using DIC for capturing structural damage in cervical cadaveric specimen within 10 ms after impact. The damage from impacts was localized at C5/6, though it was a high-speed impact. In our opinion, the quality of DIC technology it is also related to the range of motion designed for the specimens and the condition of mechanical loading.

In this study, we employed axial tensile testing, a widely recognized method for investigating cervical spine biomechanics (Zhao et al., 2020). By comparing variables such as SS, ΔIVD, ΔhIVF, ΔwIVF, and ΔLH across different segments, we observed that the DIC system could accurately measure evenly distributed changes in segment parameters. Our observation that neutral position traction resulted in more evenly distributed stress patterns in the lower cervical spine corroborates previous findings by Lin (Lin et al., 2023), thereby lending additional credence to the accuracy of this study. Another notable observation was the differential strain distribution pattern between soft tissues and bony structures. As shown in Figure 5, strain predominantly manifested in the interstitial spaces, specifically the IVD, LF, and articular processes, while bony structures exhibited minimal deformation. This pattern aligns with the therapeutic mechanisms of cervical traction, where controlled tensile forces are applied to decompress neural structures and increase intervertebral space. During mechanical loading, we observed strain concentration across multiple IVDs, articular process and laminar spaces, accompanied by increased height in these areas. These observations may provide insights into the mechanical properties of soft tissues through various measured parameters. As the IVDs undergo deformation under tensile testing, the strain experienced can lead to changes in their height, which is often measured using imaging techniques such as MRI or CT scans (Khuyagbaatar et al., 2017). Moreover, the observed elongation of the LF, indicated by changes in LH, reflects its crucial role in force distribution and neural structure protection (Dorsi et al., 2024).

These findings demonstrate DIC technology is a reliable tool for simultaneously monitoring multiple anatomical aspects and quantifying subtle deformations and provides a validated experimental framework that future researchers can build upon when studying cervical spine biomechanics and therapeutic mechanisms of cervical traction. This strain distribution pattern aligns with the therapeutic mechanisms of cervical traction, where controlled tensile forces are applied to decompress neural structures and increase intervertebral space.

To sum up, the experimental approach mentioned in this study has proven to be a reliable method for conducting biomechanical experiments on cervical cadaveric specimens *in vitro*. The DIC system effectively recorded and quantified changes in segment parameters, demonstrating its utility even under dynamic conditions. This methodology provides a validated experimental framework for future research in cervical spine biomechanics and therapeutic traction mechanisms.

### 4.5 Limitations

However, this study still has a certain limitation. In terms of specimen preparation, although fresh cadaveric specimens were used, the process of applying the speckle pattern may have induced tissue dehydration, potentially altering the viscoelastic properties of soft tissues during testing. As a surface measurement technique, DIC cannot provide direct insights into internal stress distributions or failure mechanisms occurring within the specimen under loading. The integration of DIC with finite element analysis may be able to measure the strain of overall IVD and the size of internal IVF. Furthermore, the current study was limited to tensile testing alone. The effectiveness of DIC technology in evaluating other mechanical stress modalities, such as compression and torsion, on large samples of cervical cadaveric specimens remains to be investigated. Future research should explore the applicability of DIC across various loading conditions to establish its broader utility in biomechanical testing.

## 5 Conclusion

In summary, this study applies DIC technology to explore biomechanical behaviors in cervical cadaveric specimens under dynamic conditions. The findings suggest the potential utility of DIC in cervical spine biomechanics research, offering a reliable and reproducible methodological foundation. This could significantly inform future studies aiming to broaden the application of DIC technology, potentially improving the diagnosis and treatment of cervical disorders.

## Data Availability

The raw data supporting the conclusions of this article will be made available by the authors, without undue reservation.
